# Environmental risk factors, protective factors and lifestyles for lung cancer: an umbrella review

**DOI:** 10.3389/fpubh.2025.1623840

**Published:** 2025-07-22

**Authors:** Minghao Feng, Feng Wang, Minwei Bao, Lei Zhu

**Affiliations:** ^1^Department of Thoracic Surgery, Shanghai East Hospital, School of Medicine, Tongji University, Shanghai, China; ^2^Department of Radiotherapy, Shanghai Fourth People's Hospital, School of Medicine, Tongji University, Shanghai, China; ^3^Department of Thoracic Surgery, Shanghai Pulmonary Hospital, School of Medicine, Tongji University, Shanghai, China

**Keywords:** lung cancer, environmental exposure, lifestyle factors, umbrella review, evidence grading

## Abstract

**Background:**

Lung cancer remains a leading cause of cancer-related mortality worldwide, with environmental exposures and lifestyle factors playing a crucial role in its etiology. This umbrella review aims to systematically assess and classify the strength of evidence for environmental and lifestyle factors associated with lung cancer risk.

**Methods:**

A systematic search of published meta-analyses was conducted from database inception until January 31, 2025. Eligible meta-analyses included those evaluating associations between environmental or lifestyle exposures and lung cancer risk, with effect sizes reported as risk ratio (RR), odds ratios (OR), or standardized mortality ratios (SMR). The credibility of associations was assessed using statistical significance, heterogeneity (*I*^2^), small-study effects, and excess significance bias. The evidence was categorized into convincing (Class I), highly suggestive (Class II), suggestive (Class III), and weak or non-significant associations.

**Results:**

A total of 58 meta-analyses covering 34 environmental factors and 24 lifestyle factors were included. Three environmental exposures—cadmium exposure (RR = 1.24, 95% CI: 1.18–1.29), diesel exhaust exposure (RR = 1.16, 95% CI: 1.13–1.18), and occupational exposure to paints (OR = 1.40, 95% CI: 1.29–1.51)—were classified as convincing evidence (Class I). Fifteen additional environmental factors, including secondhand smoke, benzene, formaldehyde, and indoor coal use, were classified as highly suggestive evidence (Class II). Among lifestyle factors, cooking-related exposures (OR = 1.21, 95% CI: 1.10–1.31) showed a convincing association with lung cancer risk, while dietary cholesterol intake (OR = 1.40, 95% CI: 1.20–1.64) and the Western dietary pattern (RR = 1.29, 95% CI: 1.01–1.66) were classified as highly suggestive evidence. Dietary patterns associated with reduced lung cancer risk included the Mediterranean diet (RR = 0.87, 95% CI: 0.82–0.93) and the prudent dietary pattern (RR = 0.80, 95% CI: 0.64–0.96), both of which were significantly associated with lower lung cancer risk. Heterogeneity was substantial in 48.57% of environmental associations and 39.13% of lifestyle associations, highlighting potential confounding factors.

**Conclusion:**

This umbrella review highlights multiple environmental and lifestyle exposures with strong or suggestive associations with lung cancer. These findings support stricter environmental regulations, workplace protections, and lifestyle interventions. Future research should prioritize biomarker-based exposure assessments and long-term cohort studies to refine risk estimates and inform prevention strategies.

**Systematic review registration:**

The study is registered with PROSPERO, number 1003974.

## Background

Lung cancer is one of the most common and lethal malignancies worldwide, accounting for approximately 18.7% of all cancer-related deaths (1). The incidence and mortality of lung cancer continue to increase globally, with nearly 2.5 million new cases annually, posing a major public health challenge (1, 2).

Given this burden, substantial research has focused on improving early detection and treatment strategies. However, prevention remains a key priority, particularly through identifying and mitigating modifiable risk factors. The development of lung cancer is influenced by complex interactions between genetic predisposition and environmental exposures. Well-established risk factors include air pollution, and occupational carcinogens such as paint-related exposure, asbestos and radon (3–7). Additionally, emerging evidence suggests that lifestyle factors, including dietary habits, physical activity, and household air pollution, may contribute to lung cancer risk, yet their impact remains less well characterized (8, 9).

Although many meta-analyses and systematic reviews have assessed environmental factors and lifestyles on lung cancer, most of them are inevitably restricted to a single topic. Additionally, these studies are limited by excess significance bias and publication bias. Moreover, their studies fail to establish a hierarchy of evidence among the different environmental factors and lifestyles to compare associations with lung cancer. Finally, due to the lack of clear standards, the distinctions between risk factors and protective factors become unclear. Therefore, the comprehensive and pragmatic evidence is urgent to encompasses all of these contributing factors.

To address these gaps, we synthesized evidence on environmental and lifestyle factors from existing systematic reviews and meta-analyses of observational studies, and evaluated the consistency and magnitude of this evidence, controlling for several biases in this umbrella review. We hope to provide reliable data in a comprehensive and accessible format to support clinical decision-making and guidelines.

## Methods

### Search strategy and selection criteria

We conducted a comprehensive search of PubMed, MEDLINE, Embase, and the Cochrane Database of Systematic Reviews, covering all available records up to January 31, 2025. The search strategy was provided in Supplementary material 1. We included systematic reviews that presented meta-analyses of observational studies (cohort, case-control) without language restrictions. Only meta-analyses exploring the links between potential environmental risk factors, protective factors, lifestyles and lung cancer can be included. The terms “risk factor” and “protective factor” were defined according to the WHO guidelines (Supplementary material 2).

### Inclusion and exclusion criteria

We included meta-analyses of observational epidemiological studies in humans that evaluated lifestyle and environmental (non-genetic) risk factors associated with the incidence or mortality of lung cancer. Exclusion criteria were as follows: (1) articles that did not examine environmental risk factors, environmental protective factors, or lifestyle of lung cancer; (2) articles that did not include a meta-analysis; (3) articles that did not provide sufficient data for re-analysis (e.g., individual study estimates or necessary data to calculate these); (4) non-human studies, primary studies, genetic studies, and conference abstracts; (5) meta-analyses that focused on indices of cognitive function (e.g., memory, attention, executive function, and decision-making), as these have been described elsewhere in the context of lung cancer.

When two or more meta-analyses addressed the same topic related to lung cancer, we selected only one to avoid duplication. Our first priority was to choose the meta-analysis that presented adjusted estimates over those with crude estimates. Then, we evaluated the meta-analyses based on their recency and quality using AMSTAR 2 (A Measurement Tool to Assess Systematic Reviews 2) criteria, which was used to evaluate the methodological quality of meta-analyses, focusing on criteria such as comprehensive literature search strategies, risk of bias assessment, and handling of missing data. Meta-analyses with higher AMSTAR 2 scores were prioritized because they demonstrate stricter methodological rigor, reducing the likelihood of systematic errors and enhancing the reliability of synthesized evidence. This criterion aligns with established practices in umbrella reviews to ensure evidence validity. We selected the one with the highest score. If two or more meta-analyses had the same score, we opted for the one that included more studies. This decision was based on the rationale that a greater number of studies enhances the statistical power and generalizability of the findings. If the number of studies was also equivalent, the most recent publication was prioritized to reflect the latest evidence. Some meta-analyses examined risk and protective factors, such as smoking, pollution, and diet, that might have been measured later in life, and their temporal relationship to lung cancer development may be unclear. In such cases, we included meta-analyses that focused on studies with participants diagnosed during adulthood, or created new subsets using studies with a mean age at diagnosis of 18 years or older.

### Handling of overlapping primary studies

To address the potential duplication of primary studies across different meta-analyses, we implemented a structured selection approach. When multiple meta-analyses examined the same exposure or risk factor, we selected only one to include in the umbrella review. Selection priority was based on the following criteria: (1) use of adjusted effect estimates rather than crude ones, (2) higher methodological quality as evaluated by the AMSTAR 2 tool, (3) more recent publication date, and (4) greater number of included primary studies. Furthermore, we manually compared the reference lists of overlapping meta-analyses to assess the extent of shared primary studies. In cases where overlap exceeded 50%, only the meta-analysis meeting the highest quality standard was retained. This strategy minimized double-counting and enhanced the robustness of our evidence synthesis.

### Data extraction

**Figure 1:**
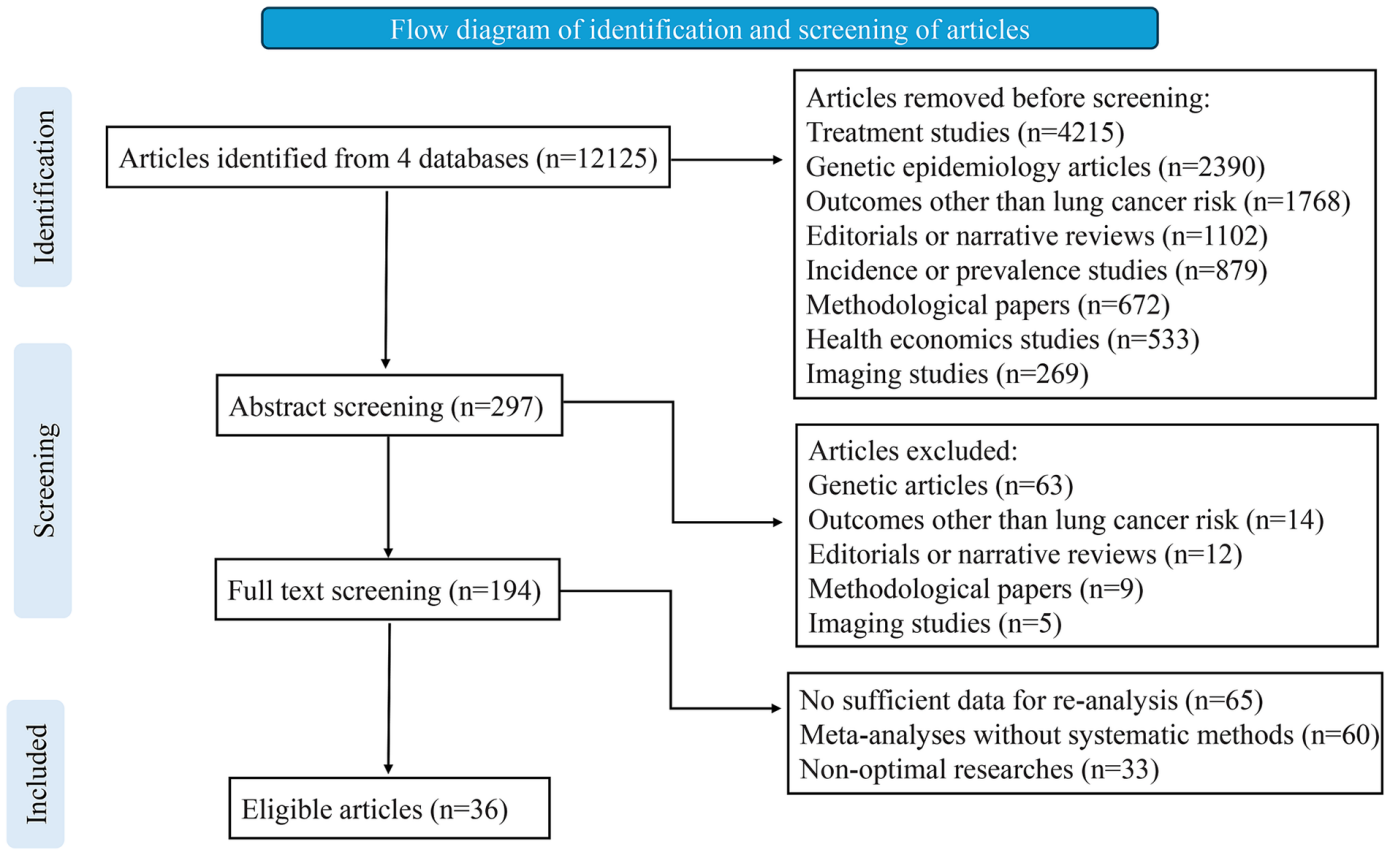
Flow chart of literature search in this umbrella review.

To identify eligible studies, two investigators (MWB and FW) independently reviewed the titles, abstracts, and full texts (Figure 1). Additionally, we manually examined the reference lists of relevant studies to find further eligible articles. Any disagreements were resolved through consultation among three authors (MWB, FW, FGY). Data extraction was carried out independently by two investigators (BT and LZ), and in the case of discrepancies, the final decision was made by discussion. For each eligible article, we recorded details such as the first author, journal, publication year, risk factors examined, and the number of studies included. When a quantitative synthesis was performed, we extracted the study-specific relative risk estimates (risk ratio [RR], odds ratio [OR], hazard ratio [HR], or standardized mortality ratio [SMR]), along with the corresponding confidence interval (CI) and the number of cases and controls for each risk factor. Adjusted estimates were prioritized over crude estimates because they account for potential confounders, providing more accurate and reliable effect size (ES) measurements. This approach minimizes bias and enhances the validity of associations reported in the meta-analyses. For studies without a quantitative synthesis, we noted a summary of the authors’ main conclusions and the reasons for not conducting a quantitative synthesis.

### Data analysis and statistics

For each meta-analysis, we calculated the overall ES and its 95% CI using both fixed-effects and random-effects models (10, 11). In addition, we computed the 95% prediction interval, which incorporates the variability between studies and provides insight into the uncertainty of the expected effect in a new study exploring the same relationship (12, 13). For the largest study in each meta-analysis, we evaluated the standard deviation (SD) of the ES and checked if it was below 0.10. If the SD was under 0.10, the difference between the effect estimates and the upper or lower 95% CI would be smaller than 0.20, implying that the uncertainty is lower than what is typically considered a small ES. For meta-analyses that included continuous data, we transformed the effect estimate to an OR using a well-established formula (14). To assess heterogeneity between studies, we used the I^2^ statistic, which represents the proportion of variation due to differences between studies (15). Generally, values above 50% are considered to reflect high heterogeneity, respectively. It is important to note that the 95% CI for *I*^2^ estimates can be wide when the number of studies is limited (16).

We examined the potential for small-study effects, which refers to the tendency for smaller studies to report larger effect sizes than larger ones, using the regression asymmetry test developed by Egger et al. (17). *p*-value below 0.10, along with a more conservative effect estimate in larger studies compared to the random-effects meta-analysis, was interpreted as indicating the presence of small-study effects.

We performed an excess statistical significance test to evaluate whether the number of studies reporting significant results (*p* < 0.05) exceeded what would be expected (18). This method examines whether the proportion of positive findings within a meta-analysis is higher than anticipated, based on the statistical power of included studies to detect plausible effects at an *α* level of 0.05. To determine the expected number of significant studies, we calculated the sum of the power estimates for each study within the meta-analysis. Since the actual ES of any meta-analysis is unknown, we used the ES from the largest study (i.e., the study with the smallest standard error) to estimate power (19). Power calculations were conducted using an algorithm based on a non-central t-distribution (20). A meta-analysis was considered to show excess statistical significance if the one-sided *p*-value was below 0.05 and the observed number of significant studies exceeded the expected count. Comparisons between observed and expected values were performed separately for each meta-analysis and were also extended to larger groups of meta-analyses by summing observed and expected values across multiple analyses.

We performed random-effects meta-analyses using credibility ceilings of 5, 10, 15, and 20% to account for possible methodological flaws in observational studies that may cause misleading significance (21, 22). All statistical analyses were conducted using two-tailed tests by the R software, version 4.0.3.

### Determining the credibility of evidence

In accordance with prior umbrella reviews, we classified the eligible meta-analyses by the strength of evidence regarding environmental risk factors, protective factors, and peripheral biomarkers associated with lung cancer into five categories: convincing (class I), highly suggestive (class II), suggestive (class III), weak (class IV), and not significant (NS) (Table 1) (23, 24). The classification criteria included *p*-values from random-effects models, the number of lung cancer cases, statistical significance of the largest study, the *I*^2^ statistic, small-study effects, excess significance bias, random-effects summary estimates with a 10% credibility ceiling, and the 95% prediction interval. For associations categorized as convincing or highly suggestive, we further assessed the evidence’s robustness by conducting subset analyses of cohort studies (both retrospective and prospective), prospective cohort studies, and studies with adjustments for at least one covariate.

**Table 1:** Level of evidence for grading levels.

	Convincing (class I)	Highly suggestive (class II)	Suggestive (class III)	Weak (class IV)	Not significant (NS)
Random effects *p* value	<0·000001	<0·000001	<0·001	<0·05	>0·05
Number of lung cancer cases	>1,000	>1,000	>1,000	–	–
P value of the largest study	<0·05	<0·05	–	–	–
Heterogeneity (*I*^2^)	<50%	–	–	–	–
Small study effects	Not detected	–	–	–	–
Excess significance bias	Not detected	–	–	–	–
95% prediction interval	Excludes the null	–	–	–	–
P value with 10% credibility ceiling	<0·05	–	–	–	–

## Results

From the inception of the database until January 31, 2025, we retrieved 10,974 articles totally, of which 36 met the eligibility criteria for inclusion. These 36 articles contributed to 58 distinct meta-analyses, covering 34 environmental factors and 24 lifestyles (Tables 2, 3). The meta-analyses focusing on environmental risk and protective factors included data from more than 1,218,149 lung cancer cases within a total population of more than 79,070,650. The median number of lung cancer cases per meta-analysis was 4,312 (IQR: 1771–12,296; range: 391–779,808), while the median total population per meta-analysis was 56,505 (IQR: 8292–597,478; range: 1652–63,312,256). Among the 10 meta-analyses derived from cohort studies, 10 from case-control studies, 14 incorporated data both from cohort, case-control or cross-sectional. The median number of study estimates per meta-analysis was 14 (IQR: 9–21; range: 3–63). Effect sizes were reported using RR, OR or SMR.

**Table 2:** Environmental risk factors and environmental protective factors of lung cancer.

	Source	Number of cases/total population	Number of study estimates	Study design	Effect metrics	Random effects summary estimate (95% CI)	Random effects *p* value	*I* ^2^	95% prediction interval	Egger p value	Large heterogeneity, small study effect, excess significance bias, or loss of significance under 10% credibility ceiling	AMSTAR 2 quality/ AMSTAR 2 quality when protocol assessment was ruled out
Convincing (class I)												
Cadmium exposure	Nawrot et al. (2015) (51)	1748/20459	3	cohort	RR	1.2 (1.18–1.29)	*p* < 0·000001	0%	1.17–1.31	0.6056	None	Moderate/low
Occupational exposure to paint (incidence)	Bachand et al. (2010) (52)	1,243/>2,416	23	Case-control	OR	1.4 (1.29–1.51)	*p* < 0·000001	43.54%	1.24–1.57	0.96	None	Low/high
Diesel exhaust exposure	Lipsett et al. (1999) (53)	3,851/NA	30	Case-control, cohort	RR	1.1 (1.13–1.18)	*p* < 0·000001	0	1.10–1.19	0.88	None	High/high
Highly suggestive (class II)												
Styrene exposure	Collins et al. (2018) (54)	2861/5979	14	Cohort, case-control	RR	1.1 (1.15–1.23)	*p* < 0·000001	85.78%	1.14–1.24	0.094	Large heterogeneity	High/moderate
Benzene exposure	Chiavarini et al. (2024) (55)	13,649/NA	21	Case-control, cohort, cross-sectional, ecological	ES	1.1 (1.07–1.27)	*p* < 0·000001	54.22%	0.80–1.54	0.906	Large heterogeneity	Moderate/moderate
Occupational exposure to paint (mortality)	Bachand et al. (2010) (52)	48,434/>NR	12	Cohort	RR	1.3 (1.34–1.41)	*p* < 0·000001	40.29%	1.33–1.43	0	small study effect, excess significance bias	Low/high
Roofers’ occupational environment	Mundt et al. (2018) (56)	1,574/>1,652	19	Cohort, case-control	ES	1.7 (1.43–2.04)	*p* < 0·000001	84.25%	0.87–3.38	0.7609	Large heterogeneity	Moderate/low
Agricultural industries environment	Lenters et al. (2009) (57)	4773/597478	17	Cohort, case-control	RR	0.62 (0.52–0.75)	*p* < 0·000001	97.9%	0.31–0.91	0.0495	Large heterogeneity, small study effect, excess significance bias, loss of significance under 10% credibility ceiling	Moderate/high
Secondhand tobacco smoke exposure in ever smokers	Kim et al. (2014) (58)	10,184/17360	12	Case-control	OR	1.27 (1.14–1.42)	*p* < 0·000001	68.39%	0.08–22.07	0.55	Large heterogeneity	Moderate/high
Secondhand tobacco smoke exposure in never smokers	Kim et al. (2014) (58)	2504/9780	18	Case-control	OR	4.79 (4.32–5.32)	*p* < 0·000001	51%	4.13–5.56	0.0144	Large heterogeneity, small study effect, excess significance bias	Moderate/high
Domestic coal use and exposure	Zhao et al. (2006) (59)	1996/5741	8	Case-control	OR	1.6 (1.39–2.02)	*p* < 0·000001	84.37%	1.28–2.18	0.3407	Large heterogeneity	Low/moderate
Air pollution-indoor coal dust	Zhao et al. (2006) (59)	1785/4220	6	Case-control	OR	2.4 (1.64–3.56)	*p* < 0·000001	97.18%	1.40–4.17	N/A	Large heterogeneity	Low/moderate
Air pollution-cooking oil vapor	Zhao et al. (2006) (59)	3304/7869	12	Case-control	OR	6.21 (2.86–13.42)	*p* < 0·000001	99.77%	1.76–20.85	0.937	Large heterogeneity	Low/moderate
Solid fuel smoke	Kurmi et al. (2012) (60)	12,419/47028	28	Case-control	OR	1.85 (1.56–2.18)	*p* < 0·000001	98.18%	1.35–2.35	0.28	Large heterogeneity	High/high
Formaldehyde	Kwak et al. (2020) (61)	11,925/>1,316,809	31	Case-control, cohort	ES	1.11 (1.08–1.13)	*p* < 0·000001	68.09%	1.07–1.14	0.4441	Large heterogeneity	Moderate/moderate
Silica dust (mortality)	Poinen-Rughooputh et al. (2016) (62)	2445/125773	63	Cohort	SMR	1.55 (1.38–1.75)	*p* < 0·000001	96.18%	1.05–1.83	0.0041	Large heterogeneity, small study effect, excess significance bias	Low/moderate
Talc exposure	Chang et al. (2017) (63)	1766/95711	14	Cohort	SMR	1.3 (1.26–1.39)	*p* < 0·000001	64.61%	1.25–1.42	0.001	Large heterogeneity, small study effect, excess significance bias, loss of significance under 10% credibility ceiling	Moderate/moderate
Residential radon exposure	Li et al. (2020) (64)	13,748/36860	28	Case-control	OR	1.5 (1.39–1.78)	*p* < 0·000001	33.68%	1.37–1.80	0.0009	small study effect	Moderate/moderate
Indoor coal use and exposure	Li et al. (2018) (65)	5647/14267	11	Case-control	OR	1.4 (1.33–1.67)	*p* < 0·000001	95.3%	0.83–2.68	0	Large heterogeneity, small study effect, excess significance bias, loss of significance under 10% credibility ceiling	Moderate/high
Suggestive (class III)												
Bitumen exposure	Mundt et al. (2017) (56)	6,695/>8,292	40	Cohort, case-control	ES	1.2 (1.11–1.40)	0.0002839	89.26%	0.70–2.20	0.9666	Large heterogeneity	Moderate/low
Occupational exposure in cotton textile workers	Lenters et al. (2009) (57)	1217/56505	11	Cohort, case-control	RR	0.72 (0.57–0.90)	0.00011	82.5%	0.55–0.98	0.0923	Large heterogeneity	Moderate/high
Tobacco smoke	Ni et al. (2018) (3)	NA	41	Case-control, cohort	OR	1.3 (1.03–1.75)	0.0281116	67%	0.76–1.92	0.7957	Large heterogeneity	High/high
Weak (class IV)												
Aromatic adducts	Gilberson et al. (2014) (66)	547/41438	4	Case-control	RR	1.3 (1.17–1.55)	*p* < 0·000001	0%	1.10–1.65	N/A	loss of significance under 10% credibility ceiling	Low/low
Silica dust (incidence)	Poinen-Rughooputh et al. (2016) (62)	391/7442	19	Cohort	SIR	1.5 (1.40–1.60)	0.00014	62.63%	0.93–2.07	0.0365	Large heterogeneity, small study effect, excess significance bias	Low/moderate
Outdoor particulate matter	Hamra et al. (2014) (4)	>41,565/5158868	18	Case-control, cohort	RR	1.0 (1.04–1.14)	0.01	53%	0.97–1.46	0.2438	Large heterogeneity	Moderate/moderate
Not significant (NS)												
Asbestos exposure in non-smokers	Ngamwong et al. (2015) (67)	7631/67450	16	Case-control, cohort	OR	1. (1.14–1.66)	0.1360371	55%	0.02–2.82	N/A	Large heterogeneity, loss of significance under 10% credibility ceiling	High/moderate
Asbestos exposure in-smokers	Ngamwong et al. (2015) (67)	7643/67504	17	Case-control, cohort	OR	1.4 (1.21–1.65)	0.3020031	74.04%	0.19–2.68	0.025	Large heterogeneity, small study effect, excess significance bias	High/moderate
Vinyl chloride exposure	Boffetta et al. (2003) (68)	612/60658	5	Case-control, cohort	SMR	0.9 (0.83–0.97)	0.1620555	38.87%	0.77–1.03	0.4	None	High/moderate
NO2	Ghassan B. Hamra (2015) (69)	43,510/3207106	15	cohort	RR	1.0 (0.98–1.13)	0.9999998	63.18%	0.94–1.16	3E-06	Large heterogeneity, small study effect, excess significance bias, loss of significance under 10% credibility ceiling	High/low
NOx	Hamra et al. (2015) (69)	3816/404260	5	cohort	RR	1.0 (1.00–1.04)	0.1117993	46.65%	0.98–1.06	0.3327	Large heterogeneity, small study effect, excess significance bias, loss of significance under 10% credibility ceiling	High/low
Living near petrochemical industrial (mortality)	Lin et al. (2017) (70)	NA/2017365	13	Cohort	RR	1.0 (0.87–1.23)	0.71	25.3%	0.56–1.91	*p* < 0·000001	small study effect, loss of significance under 10% credibility ceiling	Moderate/high
Residential petrochemical (incidence)	Lin et al. (2018) (71)	NA/466066	6	Cohort	RR	1.1 (0.74–1.92)	0.48	28.6%	0.69–2.05	0.9	loss of significance under 10% credibility ceiling	Moderate/moderate
Greenspace exposure	Zare et al. (2022) (72)	>178,858/>1,886,038	9	Cross-sectional, cohort, case-control	RR	1.0 (0.87–1.13)	0.749	0%	0.20–1.80	0.618	loss of significance under 10% credibility ceiling	High/high
Long-term ozone exposure	Sun et al. (2022) (73)	779,808/63312256	13	Cohort	RR	0.96 (0.77–1.17)	*p* < 0·000001	84.2%	0.69–1.25	0.0022	Large heterogeneity, small study effect	Moderate/moderate

AMSTAR 2: A Measurement Tool to Assess Systematic Reviews 2. HR: hazard ratio. NA: not available. OR = odds ratio. RR = relative risk. ES: effect size. SMR: standardized mortality ratio.

All statistical tests are two-tailed.

**Table 3:** Lifestyle factors of lung cancer.

	Source	Number of cases/total population	Number of study estimates	Study design	Effect metrics	Random effects summary estimate (95% CI)	Random effects *p* value	*I* ^2^	95% prediction interval	Egger *p* value	Large heterogeneity, small study effect, excess significance bias, or loss of significance under 10% credibility ceiling	AMSTAR 2 quality/AMSTAR 2 quality when protocol assessment was ruled out
Convincing (class I)												
Cooks	Bigert et al. (2015) (50)	19,370/43044	16	Case-control	OR	1.21 (1.10–1.31)	*p* < 0·000001	0%	1.07–1.35	0.48	None	Moderate/moderate
Highly suggestive (class II)												
Carotenoids intake	Gallicchio et al. (2008) (74)	4310/247706	8	Cohort	RR	0.79 (0.71–0.87)	*p* < 0·000001	0%	0.62–1.31	0.071	Loss of significance under 10% credibility ceiling	Moderate/moderate
Dietary cholesterol intake	Lin et al. (2018) (75)	8664/280320	16	Case-control, cohort	OR	1.40 (1.20–1.64)	*p* < 0·000001	58.58%	0.85–2.32	0.41	Large heterogeneity, loss of significance under 10% credibility ceiling	Low/moderate
Prudent dietary pattern	Zhao et al. (2023) (76)	3341/453049	5	Case-control, cohort	RR	0.80 (0.64–0.96)	*p* < 0·000001	59.71%	0.47–1.13	0.007	Large heterogeneity, small study effect, excess significance bias, or loss of significance under 10% credibility ceiling	Moderate/moderate
Western dietary pattern	Zhao et al. (2023) (76)	5480/457351	6	Case-control, cohort	RR	1.29 (1.01–1.66)	*p* < 0·000001	71.63%	0.83–2.01	0.885	Large heterogeneity, excess significance bias, or loss of significance under 10% credibility ceiling	Moderate/moderate
DASH	Zhao et al. (2023) (76)	16,249/790848	4	Cohort	RR	0.87 (0.77–0.98)	*p* < 0·000001	72.39%	0.63–1.19	0.953	Large heterogeneity, loss of significance under 10% credibility ceiling	Moderate/moderate
Mediterranean diet	Zhao et al. (2023) (76)	20,333/878020	10	Cohort, case-control	RR	0.87 (0.82–0.93)	*p* < 0·000001	33.79%	0.42–1.81	0.491	Loss of significance under 10% credibility ceiling	Moderate/moderate
Suggestive (class III)												
Combined healthy lifestyle	Zhang et al. (2020) (77)	14,409/1127480	8	Cohort	HR	0.76 (0.62–0.93)	*p* < 0.001	85.5%	0.51–1.12	0.079	Large heterogeneity, loss of significance under 10% credibility ceiling	Moderate/low
Fruits/vegetables pattern ^a^	Zhao et al. (2023) (76)	4931/14337	5	Case-control	RR	0.56 (0.36–0.87)	*p* < 0.001	94.91%	0.30–1.02	0.953	Large heterogeneity, loss of significance under 10% credibility ceiling	Moderate/moderate
DII	Zhao et al. (2023) (76)	8246/322828	6	Cohort	RR	1.13 (1.06–1.21)	0.000369	0%	0.82–1.57	0.208	Loss of significance under 10% credibility ceiling	Moderate/moderate
Weak (class IV)												
Beta-Carotene intake	Kordiak et al. (2022) (78)	NA/167141	8	Cohort, case-control	RR	1.16 (1.06–1.26)	0.001	0	0.16–8.19	0.58	None	Moderate/high
Citrus fruit intake	Wang et al. (2021) (79)	15,591/1471261	21	Case-control, cohort	OR	0.91 (0.84–0.98)	0.001	53.53%	0.71–1.15	0.722	Large heterogeneity, loss of significance under 10% credibility ceiling	Moderate/high
High consumption of red meat	Gnagnarella et al. (2017) (80)	3558/289840	9	Case-control, cohort	OR	1.24 (1.05–1.45)	0.0097	30.56%	0.89–1.71	0.425	excess significance bias, or loss of significance under 10% credibility ceiling	Low/moderate
Sedentary behavior	Shen et al. (2014) (81)	1321/212673	2	Cohort	RR	1.27 (1.06–1.52)	0.011	0%	1.03–1.55	0.101	None	Moderate/moderate
Not significant (NS)												
Total iron intake	Fonseca-Nunes (2014) (82)	7843/602263	3	Prospective	RR	1.12 (0.98–1.26)	0.0869869	53.6%	0–7.78	0.774	Large heterogeneity, loss of significance under 10% credibility ceiling	High/Low
High consumption of white meat	Gnagnarella et al. (2017) (80)	889/67316	4	Case-control, cohort	OR	1.04 (0.85–1.27)	0.708	0%	0.84–1.28	0.9	None	Low/moderate
High consumption of processed meat	Gnagnarella et al. (2017) (80)	2293/195451	7	Case-control, cohort	OR	1.06 (0.80–1.40)	0.681	48.5%	0.59–1.91	0.917	None	Low/moderate
High consumption of fish	Gnagnarella et al. (2017) (80)	3226/100093	9	Case-control, cohort	OR	0.93 (0.79–1.09)	0.3583	55.84%	0.65–1.33	0.418	Large heterogeneity, loss of significance under 10% credibility ceiling	Low/moderate
High consumption of offal	Gnagnarella et al. (2017) (80)	1164/4407	4	Case-control	OR	0.97 (0.80–1.18)	0.797	0%	0.78–1.20	0.869	None	Low/moderate
Traditional pattern^b^	Zhao et al. (2023) (76)	920/3452	2	Case-control	RR	1.08 (0.82–1.42)	0.33	0%	0.70–1.68	N/A	Loss of significance under 10% credibility ceiling	Moderate/moderate
Drinker patterns^c^	Zhao et al. (2023) (76)	920/3452	2	Case-control	RR	1.28 (1.03–1.59)	0.51	0%	0.82–1.99	N/A	Loss of significance under 10% credibility ceiling	Moderate/moderate
HEI^d^	Zhao et al. (2023) (76)	16,249/790848	4	Cohort	RR	0.87 (0.80–0.95)	0.16	42.28%	0.55–1.37	0.516	Loss of significance under 10% credibility ceiling	Moderate/moderate
AHEI	Zhao et al. (2023) (76)	16,249/790848	4	Cohort	RR	0.88 (0.81–0.95)	0.17	40.24%	0.67–1.15	0.661	Loss of significance under 10% credibility ceiling	Moderate/moderate
Alcohol consumption	Korte et al. (2002) (83)	9,239/>639,053	38	Case-control, cohort	RR	0.97 (0.84–1.10)	0.653	52.79%	0.75–1.19	0.344	Large heterogeneity, loss of significance under 10% credibility ceiling	High/high

AHEI: alternate Healthy Eating Index, CI: confidence interval, DASH: Dietary Approaches to Stop Hypertension, DII: dietary inflammatory index, HEI: Healthy Eating Index, OR: odds risk; RR: relative risk. a: Fruits/vegetables patterns included fruits/vegetables pattern, starchy vegetables pattern, high in vegetables and low in animal products pattern, (cooked) vegetables pattern, (salad) vegetables pattern. b: Traditional pattern was highly related to desserts, total grains, and all tubers. c: Drinker pattern was highly related to beer, wine, and hard liquor intake. d: HEI included HEI-2010 and HEI-2015. Prudent dietary pattern is defined as a dietary pattern that is high in vegetables, fruits, fish, and white meat, and low in red meat and processed foods.

Combined healthy lifestyle refers to healthy diet, body weight, physical activity, limited alcohol consumption, and avoidance of smoking. All statistical tests are two-tailed.

**Figure 2:**
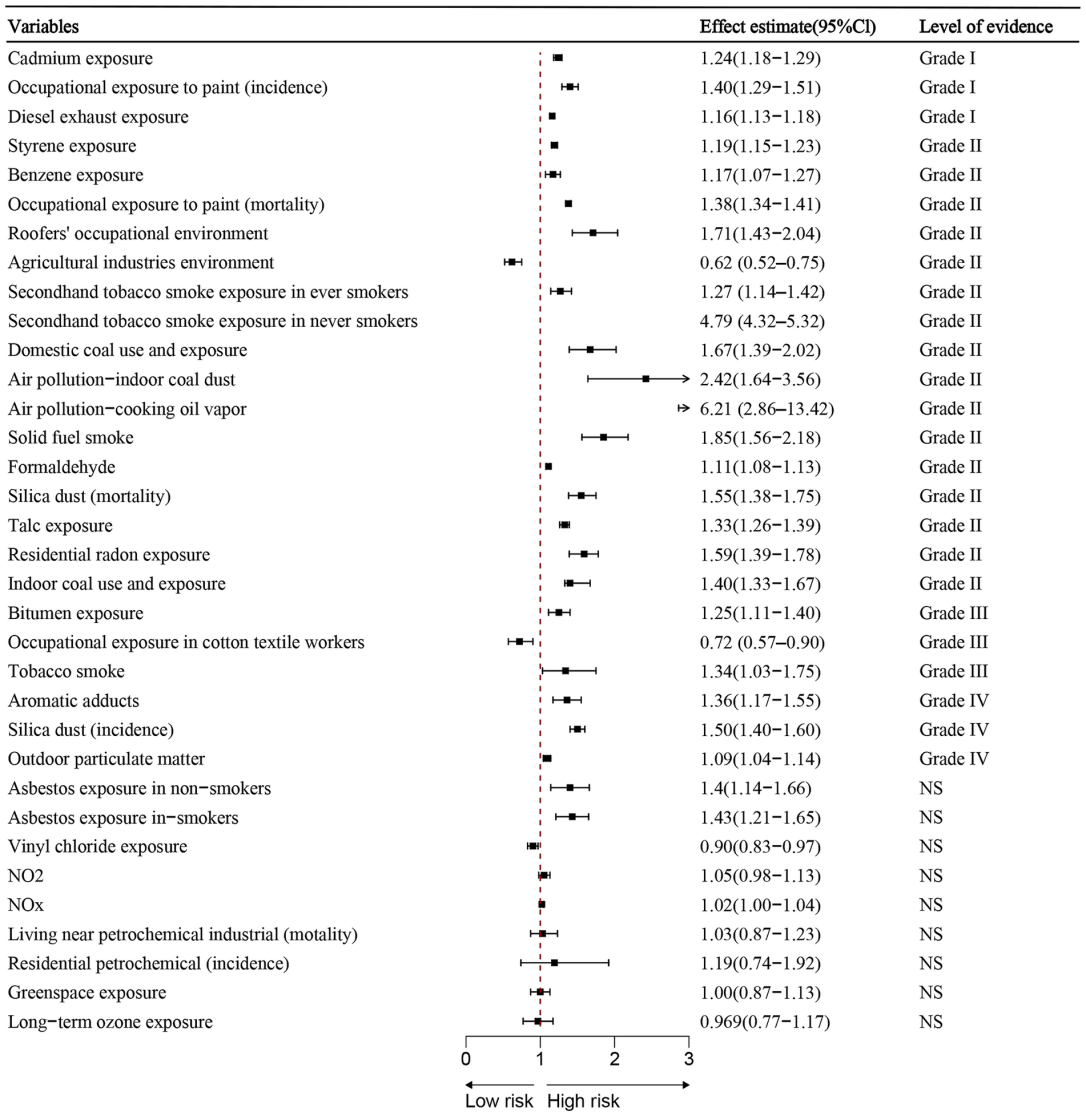
Summary estimates of environmental risk and protective factors for lung cancer. Effect sizes (RR or OR) and 95% confidence interval are shown for each meta-analysis. Factors are grouped by strength of evidence classification.

Out of the 34 associations analyzed, 26 (76.47%) showed statistical significance at *p* < 0.05, while 3 (8.82%) had *p*-values below 0.001, and 21 (61.76%) were under 0.000001. Among the statistically significant findings, 28 (82.35%) involved more than 1,000 lung cancer cases per association. Substantial heterogeneity (*I*^2^ > 50%) was present in 16 (47.06%) associations. Additionally, 11 (32.35%) remained significant after accounting for small-study effects and excess significance bias. The 95% prediction interval excluded the null in 17 (50%) associations, and 24 (70.59%) retained statistical significance under a 10% credibility ceiling (Table 2; Figure 2).

The 24 meta-analyses focused on lifestyles involved data from 184,795 lung cancer cases and more than 9,953,081 population totally. The median number of lung cancer cases per meta-analysis was 5,205 (IQR: 2050–15,755; range: 889–20,333), while the median total population per meta-analysis was 280,320 (IQR: 67316–790,848; range:3452–1,471,261). Among the 7 meta-analyses derived from cohort studies, 5 from case-control studies, 12 incorporated data both from case-control, cohort, cross-sectional or prospective studies. The median number of study estimates per meta-analysis was 6 (IQR: 4–9; range: 2–38). Effect sizes were reported using RR, OR, or HR.

**Figure 3:**
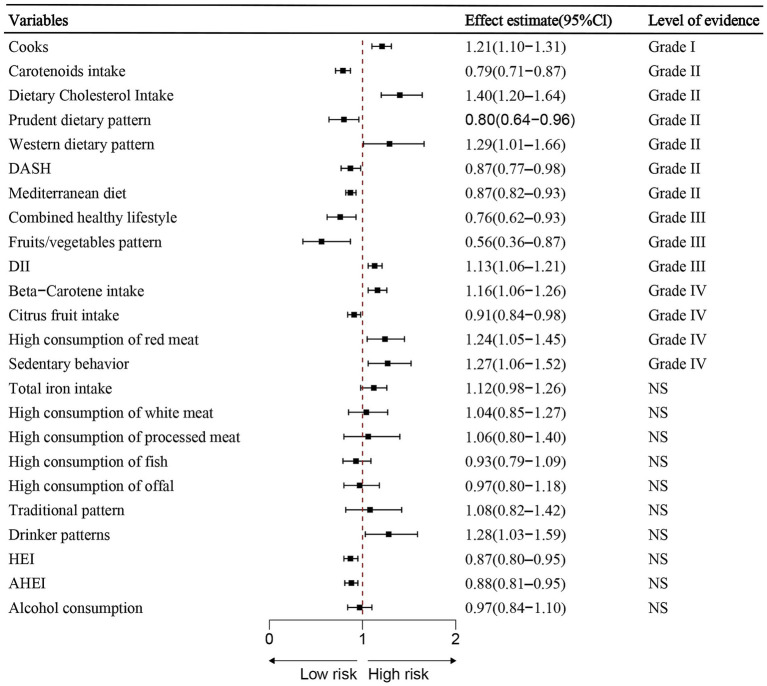
Summary estimates of lifestyle-related risk and protective factors for lung cancer. Associations are ordered by effect size and evidence class. Error bars indicate 95% confidence interval.

Among the 24 associations, 14 (58.33%) were statistically significant under a random-effects model, with three (12.50%) showing *p*-values less than 0.001 and seven (29.17%) reporting p-values below 0.000001. 12 (92.31%) of these statistically significant associations involved over 1,000 lung cancer cases. A large degree of heterogeneity (*I*^2^ > 50%) was observed in 10 (41.67%) of the associations. Additionally, only one (7.14%) of the 14 associations showed statistical significance without small study effects or excess significance bias. The 95% prediction interval excluded the null hypothesis in 17 (70.83%) of the associations, and 6 (25%) maintained significance under a 10% credibility ceiling (Table 3; Figure 3).

AMSTAR 2 quality assessments were performed for all associations. Among the 34 meta-analyses on environmental risk factors and protective factors, 10 (29.41%) were rated as high quality, 16 (47.06%) as moderate, and 8 (23.53%) as low (Table 2). After excluding the protocol criterion, 7 (20.59%) were classified as low. For the 24 meta-analyses on lifestyles, two (8.33%) were rated as high quality, 16 (66.67%) were rated as moderate (Table 3). Once the protocol requirement was removed, 22 (91.67%) of the studies were rated as high or moderate quality.

Environmental risk factors classified by strength of evidence:

Convincing evidence (Class I):

- Cadmium exposure (RR = 1.24, 95% CI: 1.18–1.29).
- Painter environment (incidence) (OR = 1.40, 95% CI: 1.29–1.51).
- Diesel exhaust exposure (RR = 1.16, 95% CI: 1.13–1.18).

Highly suggestive evidence (Class II, n = 15):

- Styrene exposure (RR = 1.19, 95% CI: 1.15–1.23).
- Benzene exposure (ES = 1.17, 95% CI: 1.07–1.27).
- Painter environment (mortality) (RR = 1.38, 95% CI: 1.34–1.41).
- Roofers environment (RR = 1.71, 95% CI: 1.43–2.04).
- Secondhand tobacco (ever smokers) (OR = 1.27, 95% CI: 1.14–1.42).
- Secondhand tobacco (never smokers) (OR = 4.79, 95% CI: 4.32–5.32).
- Domestic coal environment (OR = 1.67, 95% CI: 1.39–2.02).
- Indoor coal dust (air pollution) (OR = 2.42, 95% CI: 1.64–3.56).
- Cooking oil vapor (OR = 6.21, 95% CI: 2.86–13.42).
- Solid fuel smoke (OR = 1.85, 95% CI: 1.56–2.18).
- Formaldehyde exposure (ES = 1.11, 95% CI: 1.08–1.13).
- Silica dust (mortality) (SMR = 1.55, 95% CI: 1.38–1.75).
- Talc exposure (SMR = 1.33, 95% CI: 1.26–1.39).
- Residential radon exposure (OR = 1.59, 95% CI: 1.39–1.78).
- Indoor coal use (OR = 1.40, 95% CI: 1.33–1.67).

Protective environmental factors:

- Agricultural industries environment (RR = 0.62, 95%CI:0.52–0.75) classified as convincing.
- Cotton textile exposure (RR = 0.72, 95%CI: 0.57–0.90) classified as suggestive evidence (Class III).

Suggestive evidence (Class III) – Risk factors: Bitumen exposure and tobacco smoke.

Weak evidence (Class IV): Aromatic adducts and silica dust (incidence) and outdoor particulate matter

Lifestyle factors classified by strength of evidence:

Convincing evidence (Class I – Risk): Cooking (OR = 1.21, 95% CI: 1.10–1.31)

Highly suggestive evidence (Class II):

- Risk factors:
  Dietary cholesterol intake (OR = 1.40, 95% CI: 1.20–1.64).
  Western dietary pattern (RR = 1.29, 95% CI: 1.01–1.66).
- Protective factors:
  Carotenoids intake (RR = 0.79, 95% CI: 0.71–0.87).
  Prudent dietary pattern (RR = 0.80, 95% CI: 0.64–0.96).
  DASH diet (RR = 0.87, 95% CI: 0.77–0.98).
  Mediterranean diet (RR = 0.87, 95% CI: 0.82–0.93).

Suggestive evidence (Class III):

- Protective factors:
  Combined healthy lifestyle (HR = 0.76, 95% CI: 0.62–0.93).
  Fruits/vegetables pattern (RR = 0.56, 95% CI: 0.36–0.87).
- Risk factor: dietary inflammatory lifestyle (OR = 1.13, 95% CI: 1.06–1.21).

Weak evidence (Class IV):

- Protective factor: Citrus fruit intake.
- Risk factors: Beta-carotene intake, high consumption of red meat and sedentary behavior.

Among the 34 environmental factor meta-analyses, 11 (32.35%) showed significant small-study effects or excess significance bias, whereas this was observed in only 1 (7.14%) of the 14 statistically significant lifestyle-related associations. However, when taking into account the total number of lifestyle analyses (n = 24), the relative proportion of associations affected by small-study effects or excess significance bias rose to 8.33 and 12.5%, respectively. These biases were more commonly observed in lifestyle-related analyses that included a smaller number of primary studies and had higher heterogeneity.

## Discussion

Our umbrella review systematically assessed the associations between environmental and lifestyle factors and lung cancer risk, synthesizing data from 58 meta-analyses covering 34 environmental exposures and 24 lifestyle-related factors. Our findings provide a comprehensive evaluation of existing evidence, identifying robust associations while addressing methodological concerns such as heterogeneity and potential biases.

Among environmental risk factors, three exposures—cadmium, occupational exposure to diesel exhaust, and paint-related environments—were classified as convincing evidence (Class I). These results align with prior studies demonstrating the carcinogenic effects of heavy metals and industrial pollutants (25, 26). Additionally, 15 environmental factors were categorized as highly suggestive evidence (Class II), including styrene, benzene, secondhand tobacco smoke, domestic coal exposure, and air pollution-related factors (indoor coal dust, solid fuel smoke, and cooking oil vapor). These findings reinforce the established role of air pollution and chemical exposures in increasing lung cancer risk (27, 28).

Cadmium is a toxic heavy metal commonly found in industrial emissions, cigarette smoke, and contaminated food sources, and has been classified as a Group 1 carcinogen by the International Agency for Research on Cancer (IARC) (29). Our findings reinforce cadmium as a convincing lung cancer risk factor (Class I, RR = 1.24, 95% CI: 1.18–1.29), consistent with prior occupational cohort studies linking cadmium exposure to increased lung cancer incidence (30, 31). The primary carcinogenic mechanisms involve oxidative stress, DNA damage, inhibition of DNA repair pathways, and chronic inflammation (32). Cadmium also disrupts cellular homeostasis by interfering with zinc-dependent enzymes, leading to epigenetic modifications such as DNA methylation changes and histone modifications (33). This could explain its long-term carcinogenic effects even after exposure cessation. Given these mechanisms, targeting cadmium-induced epigenetic alterations could be a potential avenue for chemopreventive strategies (34). Although regulatory measures have reduced cadmium emissions in some countries, industrial workers and populations consuming high levels of cadmium-contaminated food remain at elevated risk.

Diesel exhaust is a well-documented carcinogen composed of fine particulate matter (PM2.5), polycyclic aromatic hydrocarbons (PAHs), and nitrogen oxides, all of which contribute to lung cancer risk (35)^.^ Our analysis confirms a significant association between occupational diesel exhaust exposure and lung cancer risk (RR = 1.16, 95% CI: 1.13–1.18). The carcinogenic mechanisms involve chronic inflammation, oxidative DNA damage, and mutagenesis due to PAHs binding to DNA, forming bulky adducts (36). In addition, PM2.5 particles penetrate deep into the alveoli, inducing persistent inflammation and cell proliferation, both of which contribute to tumorigenesis (37). However, clean diesel technology (low-emission diesel) has been increasingly adopted, reducing overall emissions and potentially lowering risk in recent decades. Despite these improvements, workers in transportation, mining, and construction industries still experience prolonged exposure to diesel exhaust, warranting enhanced protective measures. Our umbrella review did not explore this time-dependent trend, which remains an important area for future research.

Occupational exposure to paint-related chemicals has long been associated with an increased risk of lung cancer, as evidenced by our study’s finding of a significant association (OR = 1.40, 95% CI: 1.29–1.51). Painters are routinely exposed to organic solvents, heavy metals, and volatile chemicals such as benzene, toluene, and formaldehyde, which have been implicated in carcinogenesis through oxidative DNA damage and immune suppression (38). However, some European studies have found that since lead-based paints have been gradually phased out, the lung cancer risk among painters has declined (39). Our analysis did not differentiate between different generations of paint formulations, which may lead to an overestimation of the current risk for modern painters. Future studies should account for changes in industrial safety regulations and variations in exposure levels across different time periods to refine risk assessments. Meanwhile, protective measures, such as improved ventilation, use of respirators, and transition to safer water-based paints, should be prioritized to mitigate ongoing risks.

Cooking-related exposures, particularly from high-temperature cooking methods such as frying, stir-frying, and grilling, have been associated with lung cancer risk, especially among non-smokers (40). Our analysis supports this association, identifying cooking oil fumes and solid fuel smoke as significant contributors. The primary concern is the release of PAHs and aldehydes, which can induce DNA damage, inflammation, and epigenetic modifications leading to carcinogenesis (41). Notably, in Western countries, cooking exposure has not shown strong associations with lung cancer, likely due to differences in cooking practices (e.g., lower-temperature baking), better kitchen ventilation, and reduced reliance on solid fuels (42). This difference may be due to the lack of stratification by cooking methods in our study, which could have influenced the estimated risk in Western populations. Public health initiatives should focus on promoting proper ventilation, use of range hoods, and alternative cooking methods to minimize household exposure. Two recent studies further support our findings. Zhou et al. reported persistent lung cancer burden from household PM 2.5 in low- and middle-income countries, reinforcing regional disparities (43). Wang et al. identified cadmium-related DNA methylation markers linked to lung cancer risk, especially among nonsmokers (44). These studies highlight the value of updated exposure assessments and molecular biomarkers in complementing meta-analytic evidence on environmental carcinogenesis.

Temporal dynamics in exposure levels also warrant careful consideration. Many of the environmental risk factors evaluated in our review—such as cadmium, diesel exhaust, and occupational exposure to paint—have been subject to evolving regulatory policies and technological improvements over recent decades. For example, the adoption of low-emission diesel engines and the removal of lead-based paint have substantially reduced population-level exposure in many countries (45). However, most included meta-analyses did not conduct stratified analyses by time period, potentially conflating historical high-exposure data with more recent, lower-exposure environments. This limitation underscores the need for future meta-analyses to incorporate temporal meta-regression or subgroup analyses by exposure period to better reflect real-world contemporary risks and guide policymaking (46).

Notably, small-study effects and excess significance bias were more frequently observed in lifestyle-related associations than in environmental factors. This pattern may reflect lower statistical power, selective reporting, or heterogeneity in lifestyle studies, which often rely on self-reported data and vary greatly in exposure definitions. These findings suggest that while some lifestyle factors (e.g., cooking-related exposures, cholesterol intake) demonstrated convincing or highly suggestive evidence, caution is warranted in interpreting results from smaller or heterogeneous lifestyle meta-analyses. In contrast, environmental exposures with large occupational cohorts and more standardized exposure metrics exhibited greater methodological consistency.

In addition to the meta-analytic findings, broader considerations of gene–environment interactions and exposure disparities deserve attention. Although the current umbrella review focused on observational evidence, individual genetic susceptibility, such as polymorphisms in detoxification enzymes or DNA repair genes, may substantially modulate the effects of environmental exposures on lung cancer risk (47, 48). Future research integrating genetic profiling with exposure data could help identify high-risk subgroups and inform personalized prevention. Vulnerable populations, including non-smoking women, the elderly, and workers in high-exposure occupations, often face disproportionate disease burdens (49). Moreover, disparities are especially pronounced in low-resource settings, where household air pollution from biomass fuels, poor ventilation, and limited occupational safety measures elevate exposure levels (50). These disparities must be addressed to ensure equitable risk reduction and global health protection.

Despite the strengths of this study, several limitations must be acknowledged. First, heterogeneity across meta-analyses remains a concern due to differences in study designs, exposure assessment methods, and population demographics. Second, time trends in risk factors were not fully explored, particularly regarding clean diesel technology and regulatory changes in industrial exposures. Third, residual confounding may influence the observed associations, as factors such as smoking, socioeconomic status, and genetic susceptibility were not uniformly adjusted for across studies. Fourth, current exposure assessment methods rely largely on self-reported data, which may introduce measurement bias. Future research should incorporate biomarker-based studies (e.g., urinary cadmium levels, PAH-DNA adducts in diesel-exposed workers) to improve exposure accuracy. Additionally, gene–environment interaction studies should be conducted to identify high-risk subgroups with genetic susceptibility to environmental carcinogens. Future research should also consider exposome-based approaches that integrate external, internal, and behavioral exposures across the life span. Such frameworks may better capture the complexity and timing of risk factors contributing to lung cancer and support more precise and personalized prevention strategies. Finally, longitudinal studies should assess the impact of evolving regulatory measures on lung cancer risk, particularly in industries undergoing technological transitions.

## Conclusion

This umbrella review confirms strong associations between cadmium, diesel exhaust, and paint-related occupational exposures with lung cancer risk. Cooking-related exposures also contribute, particularly in non-smokers. While several exposures demonstrated robust evidence, others require further investigation to clarify mechanisms and interactions. Future research should integrate cohort studies and biomarker-based assessments to improve exposure characterization. Public health efforts should prioritize reducing exposure to established carcinogens to mitigate the global burden of lung cancer.

## Data Availability

The original contributions presented in the study are included in the article/Supplementary material, further inquiries can be directed to the corresponding author.
