# Supplementary material for: Environmental risk factors, protective factors and lifestyles for lung cancer: an umbrella review
Source: Front Public Health. 2025 Jul 22;13:1623840. doi: 10.3389/fpubh.2025.1623840 (PMC12321780; doi:10.3389/fpubh.2025.1623840)
Supplement: Supplementary file 2 [file Table_1.docx]

**Supplementary material 1 Search strategy for this study**

1. PubMed search strategy

(“Lung Neoplasms”[MeSH] OR “lung cancer” OR “pulmonary cancer” OR “lung carcinoma” OR “NSCLC” OR “SCLC”)

AND

(“Environmental Exposure”[MeSH] OR “Air Pollution”[MeSH] OR “Occupational Exposure”[MeSH] OR “environmental risk factors” OR “cadmium” OR “diesel exhaust” OR “paint exposure” OR “asbestos” OR “radon” OR “air pollution” OR “benzene” OR “formaldehyde” OR “coal dust” OR “indoor air pollution” OR “secondhand smoke”)

AND

(“Life Style”[MeSH] OR “lifestyle factors” OR “diet” OR “physical activity” OR “smoking” OR “sedentary behavior” OR “alcohol consumption” OR “Western diet” OR “Mediterranean diet” OR “prudent diet” OR “cooking oil fumes” OR “solid fuel smoke” OR “red meat consumption” OR “dietary cholesterol” OR “carotenoids intake”)

AND

(“Meta-Analysis”[Publication Type] OR “Systematic Review”[Publication Type] OR “Review”[Publication Type])

AND

("humans"[MeSH] AND English[lang])

2. Embase search strategy

('lung cancer'/exp OR 'lung neoplasm' OR 'pulmonary carcinoma' OR 'non-small cell lung cancer' OR 'small cell lung cancer')

AND

('environmental exposure'/exp OR 'occupational exposure'/exp OR 'air pollution'/exp OR 'cadmium' OR 'diesel exhaust' OR 'paint exposure' OR 'asbestos' OR 'radon' OR 'benzene' OR 'formaldehyde' OR 'coal dust' OR 'indoor air pollution' OR 'secondhand smoke')

AND

('life style'/exp OR 'dietary intake'/exp OR 'physical activity'/exp OR 'smoking'/exp OR 'alcohol consumption'/exp OR 'Western diet' OR 'Mediterranean diet' OR 'prudent diet' OR 'cooking oil fumes' OR 'solid fuel smoke' OR 'red meat consumption' OR 'dietary cholesterol' OR 'carotenoids intake')

AND

('meta analysis'/exp OR 'systematic review'/exp OR 'review'/exp)

AND

([humans]/lim AND [english]/lim)

3. Cochrane Database of Systematic Reviews search strategy

Lung Neoplasms OR Lung Cancer OR Pulmonary Cancer OR NSCLC OR SCLC

AND

Environmental Exposure OR Air Pollution OR Occupational Exposure OR Cadmium OR Diesel Exhaust OR Paint Exposure OR Asbestos OR Radon OR Benzene OR Formaldehyde OR Coal Dust OR Indoor Air Pollution OR Secondhand Smoke

AND

Life Style OR Dietary Intake OR Physical Activity OR Smoking OR Alcohol Consumption OR Western Diet OR Mediterranean Diet OR Prudent Diet OR Cooking Oil Fumes OR Solid Fuel Smoke OR Red Meat Consumption OR Dietary Cholesterol OR Carotenoids Intake

AND

Meta-Analysis OR Systematic Review OR Review

AND

Humans AND English Language

**Supplementary material 2: Definition of “Risk Factor” and “Protective Factor” According to WHO Guidelines**

1. Definition of “Risk Factor”

According to the World Health Organization (WHO), a risk factor is defined as:

"Any attribute, characteristic, or exposure of an individual that increases the likelihood of developing a disease or injury."

For the purposes of this study, risk factors for lung cancer are categorized as follows:

(a) Environmental Risk Factors

- Air pollution (e.g., particulate matter (PM2.5), nitrogen dioxide (NO₂), ozone)
- Occupational exposures (e.g., asbestos, radon, diesel exhaust, benzene, formaldehyde, silica dust, paint-related chemicals)
- Household exposures (e.g., secondhand tobacco smoke, indoor coal use, cooking oil fumes, solid fuel smoke)
- Heavy metal exposure (e.g., cadmium, arsenic, lead)
- Industrial chemical exposure (e.g., styrene, polycyclic aromatic hydrocarbons (PAHs), bitumen, aromatic adducts)

(b) Lifestyle Risk Factors

- Tobacco use (e.g., active smoking, secondhand smoke exposure)
- Dietary factors (e.g., high dietary cholesterol intake, Western dietary pattern, high consumption of red meat)
- Physical inactivity and sedentary behavior
- Alcohol consumption

Risk factors identified in this study were classified based on epidemiological evidence and were categorized as convincing, highly suggestive, suggestive, or weak evidence according to predefined statistical criteria.

2. Definition of “Protective Factor”

The WHO defines a protective factor as:

"Any attribute or characteristic that reduces the probability of occurrence of a disease or adverse health outcome."

For this study, protective factors against lung cancer include:

(a) Environmental Protective Factors

- Occupational exposure with potential protective effects (e.g., cotton textile work, agricultural industry exposure)

(b) Lifestyle Protective Factors

- Healthy dietary patterns (e.g., Mediterranean diet, prudent dietary pattern, Dietary Approaches to Stop Hypertension (DASH) diet)
- Antioxidant and nutrient-rich diets (e.g., high intake of carotenoids, fruits, vegetables)
- Active lifestyle (e.g., regular physical activity, reduced sedentary behavior)

Protective factors were identified based on available meta-analyses and categorized into different levels of evidence following the same credibility assessment applied to risk factors.
